# GMP-Compliant Isolation and Large-Scale Expansion of Bone Marrow-Derived MSC

**DOI:** 10.1371/journal.pone.0043255

**Published:** 2012-08-14

**Authors:** Natalie Fekete, Markus T. Rojewski, Daniel Fürst, Ludwika Kreja, Anita Ignatius, Julia Dausend, Hubert Schrezenmeier

**Affiliations:** 1 Institut für Transfusionsmedizin, Universitätsklinikum Ulm, Ulm, Germany; 2 Institut für klinische Transfusionsmedizin und Immungenetik, Ulm, Ulm, Germany; 3 Institut für Unfallchirurgische Forschung und Biomechanik, Ulm, Germany; University of Medicine and Dentistry of New Jersey, United States of America

## Abstract

**Background:**

Mesenchymal stromal cells (MSC) have gained importance in tissue repair, tissue engineering and in immunosupressive therapy during the last years. Due to the limited availability of MSC in the bone marrow, *ex vivo* amplification prior to clinical application is requisite to obtain therapeutic applicable cell doses. Translation of preclinical into clinical-grade large-scale MSC expansion necessitates precise definition and standardization of all procedural parameters including cell seeding density, culture medium and cultivation devices. While xenogeneic additives such as fetal calf serum are still widely used for cell culture, its use in the clinical context is associated with many risks, such as prion and viral transmission or adverse immunological reactions against xenogeneic components.

**Methods and Findings:**

We established animal-free expansion protocols using platelet lysate as medium supplement and thereby could confirm its safety and feasibility for large-scale MSC isolation and expansion. Five different GMP-compliant standardized protocols designed for the safe, reliable, efficient and economical isolation and expansion of MSC was performed and MSC obtained were analyzed for differentiation capacity by qPCR and histochemistry. Expression of standard MSC markers as defined by the International Society for Cellular Therapy as well as expression of additional MSC markers and of various chemokine and cytokine receptors was analysed by flow cytometry. Changes of metabolic markers and cytokines in the medium were addressed using the LUMINEX platform.

**Conclusions:**

The five different systems for isolation and expansion of MSC described in this study are all suitable to produce at least 100 millions of MSC, which is commonly regarded as a single clinical dose. Final products are equal according to the minimal criteria for MSC defined by the ISCT. We showed that chemokine and integrin receptors analyzed had the same expression pattern, suggesting that MSC from either of the systems show equal characteristics of homing and adhesion.

## Introduction

MSC present an exciting prospect for fulfilling previously unmet needs in cell therapy, tissue repair, tissue engineering and gene therapy. Translational research involving MSC aims at developing an off-the-shelf cellular therapeutic product tailored to a myriad of clinical scenarios.

As per the definition of the ISCT [1], MSC have to be plastic-adherent when maintained *in vitro* and must be able to differentiate into osteoblasts, adipocytes and chondroblasts following standard cell culture differentiating conditions. In addition, ≥95% the MSC population has to express CD73, CD90 and CD105 and must lack expression of hematopoietic markers such as CD14, CD34, CD45 and HLA-DR [1].

Due to the low content of primary MSC in the bone marrow (0.001–0.01% of total nucleated cells [2]), significant *ex vivo* cell amplification prior to clinical application is strictly necessary to obtain therapeutic applicable cell doses of 1–5 millions cells/kg body weight [3].

While more than 100 MSC-related clinical trials are currently registered at www.clinicaltrials.gov, a considerable variation can be observed in the mode and stringency regarding the production of MSC. To date, no infusional toxicity or immediate adverse effects have been observed [4], substantiating the safety and feasibility of MSC as therapeutic agents. Despite the great interest in MSC for a variety of clinical indications, defined and universally accepted standards and controls for their large-scale clinical production are still lacking.

Translation of preclinical investigation from the bench into clinical-grade large-scale MSC expansion to the patient’s bedside necessitates precise definition and standardization of all procedural parameters including the source, collection methods, cell seeding density, culture medium and cultivation devices.

Accordingly, MSC are now considered as advanced therapy medicinal products (ATMPs) by the European Medicines Agency (EMA) by regulation No. [EC] 1394/2007 of the European Commission) [5] and must be produced in compliance with Good Manufacturing Practices (GMP) to ensure reproducibility, efficacy and safety of the therapeutic product. The defined GMP standards ensure that cells are produced under highest standards of sterility, quality control and documentation following a standard operating procedure. While xenogeneic additives such as fetal calf serum (FCS) is still widely utilized for cell culture and tolerated by the regulatory authorities, its use in the clinical context is associated with many risks, such as prion and viral transmission or adverse immunological reactions against xenogeneic components. Hence, we established an animal-free expansion protocol using the hemoderivate platelet lysate as a medium supplement and could confirm its safety and feasibility for large-scale MSC isolation and expansion for clinical application [6]. Having eliminated animal products from our cultivation system, we have further optimized the procedural-steps and hereby present five GMP-compliant standardized protocols designed for the safe, reliable, efficient and economical isolation and expansion of MSC from the bone marrow for clinical application.

## Methods

The project was approved by the Ethical Committee of Ulm University after obtaining written consent.

### Bone Marrow Collection

Bone marrow (BM) aspirates were either purchased from LONZA (Gaithersburg, USA) (n = 5) or aspiration from iliac crest of a maximum of 37 mL of BM was performed from healthy volunteer donors at the IKT Ulm (25 aspirations from 21 individual donors) using up to four 20 mL syringes (Omnifix, Germany) and transplant aspiration needles (Somatex, Germany) after informed consent according to the Declaration of Helsinki. Each syringe was prefilled with 1,000 I.U. of heparin (B.Braun, Melsungen, Germany or ratiopharm, Neu-Ulm, Germany) in 2 mL of NaCl (B.Braun, Meslungen, Germany). BM-donors were tested according to guidelines for the preparation of blood and blood components and the use of blood products (Hemotherapy Guidelines) according to §§ 12 and 18 of the German transfusion law (Richtlinien zur Gewinnung von Blut und Blutbestandteilen und zur Anwendung von Blutprodukten (Hämotherapie). [7].

### Cell Count of BM

The amount of cells was acquired by using a SYSMEX KX-21N system (Sysmex, Norderstedt, Germany). Number of mononuclear cells per µL was calculated: number of mononuclear cells per µL [cells/µL] =  number of leucocytes per µL [cells/µL]×(100 [%] – percentage of neutrophiles [%]).

### Isolation and Expansion of Bone Marrow-derived Human MSC

A single-step protocol as well as four different two-step protocols were tested in this study. The characteristics of these protocols are listed in **Table 1**.

**Table 1:** Characteristics of the different expansion protocols.

System	single-step	two-step			
Protocol	SSP	TSP1	TSP2	TSP3	TSP4
**Passage 0**					
Preferred culture system	5 chamber stack	2 chamber stack	2 chamber stack	2 chamber stack	2 chamber stack
Cells seeded/cm^2^	12,000 MNC	50,000 WBC	50,000 WBC	50,000 WBC	50,000 WBC
Culture time in days	10–14	14	10	14	10
% PL as supplement	10	5	5	10	10
**Passage 1**					
Preferred culture system	n/a	2 chamber stack	2 chamber stack	2 chamber stack	2 chamber stack
MSC seeded/cm^2^	n/a	4000	4000	4000	4000
Culture time In days	n/a	7	5	7	5
% PL as supplement	n/a	8	8	10	10

PL: platelet lysate.

#### Single-step-protocol

Complete medium (CMSSP) consisted of alphaMEM (Lonza, Basel, Switzerland), supplemented with 10% platelet lysate (IKT Ulm, Ulm, Germany – for detailed production process see [6]); 2 i.u Na-heparin per mL complete medium were added. Cultures were performed without the use of antibiotics. We developed all our protocols on purpose without antibiotics to minimize the exposure of patients to substances of non-human origin and to avoid false-negative results of bacterial testing by antibiotics in the culture medium. Here, we demonstrate feasibility of this approach.

Unprocessed bone marrow was seeded in a first step at a density of 12.000 mononuclear cells (MNC)/cm^2^ on 5-chamber CellStacks (Corning, Amsterdam, The Netherlands) in 750 mL of CMSSP. After 3 days, supernatant containing non-adherent cells was removed, cells were rinsed with PBS without Ca^2+^/Mg^2+^ (Lonza, Basel, Switzerland) and 700 mL of CMSSP was added. Partial exchange of 300 mL of CMSSP was performed twice weekly. After 10 to 14 days, cells were rinsed with PBS and harvested using TRYPZEAN (Lonza, Basel, Switzerland).

#### Two-step-protocols

**Figure 1:**
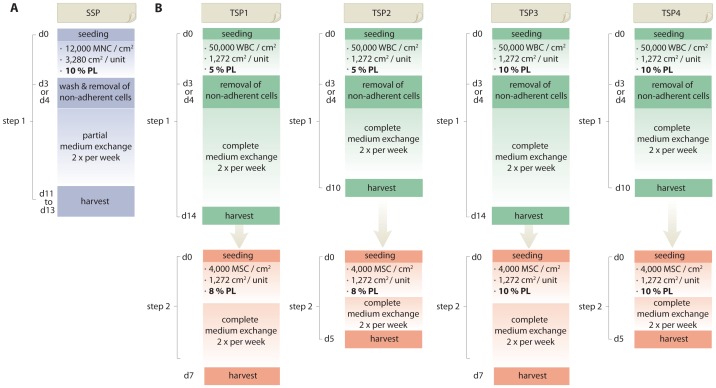
Schematic summary of the GMP-compliant single-step and two-step protocols used for clinical-scale isolation and expansion of MSCs from BM.

Unprocessed bone marrow was seeded in a first step at a density of 50.000 white blood cells (WBC)/cm^2^ on 2-chamber CellStacks (Corning, Amsterdam, The Netherlands) in 300 mL of alphaMEM, supplemented with either 5% platelet lysate and 1 i.u Na-heparin per mL final concentration (CMTS-5) for protocol options TSP1 and TSP2 or 10% platelet lysate (PL) and 1 i.u. Na-heparin per mL final concentration (CMTS-10) for protocol options TSP3 and TSP4 (**Figure 1**). Cultures were performed without the use of antibiotics. After 3 days, supernatant with non-adherent cells was removed and 300 mL of freshly prepared CMTS-5 or CMTS-10 was added to the corresponding 2-chamber CellSTACKS. Complete exchange of medium was performed twice per week. After either 10 days (for TSP2 and TSP4) or after 14 days (for TSP1 and TSP3) cells were rinsed with PBS and harvested using TRYPZEAN. These passage 0 cells were seeded at a density of 4.000 MSC/cm^2^ on 2-chamber CellSTACKS in 8% platelet lysate and 1 i.u Na-heparin per mL final concentration (CMTS-8) for protocol options TSP1 and TSP2 or in CMTS-10 for protocol options TSP3 and TSP4. Complete exchange of medium was performed twice per week. After additional 5 days (for TSP2 and TSP4) or 7 days (for TSP1 and TSP3) cells were rinsed with PBS and harvested using TRYPZEAN.

For further passages, cells were grown in medium containing PL in the same concentration as in passage 1.

#### Colony forming units (CFU-F) assay

CFU-F were set up by seeding non-manipulated BM at the same density as the main culture (i.e. 12.000 MNC/cm^2^ for single-step protocols and 50.000 WBC/cm^2^ for two-step protocols) in alpha-MEM (LONZA, Basel, Switzerland) supplemented with 10% of PL. Cultures were performed without the use of antibiotics. Non-adherent cells were removed at the same time as in the corresponding main culture. For single-step cultures, cells were also rinsed using Ca^2+^/Mg^2+^ (LONZA, Basel, Switzerland). Complete (single-step protocol) or partial (two-step protocols) medium exchange was performed twice per week. Colonies consisting of more than 5 cells were counted after 7 days of culture to determine clonogenicity. The early counting of colonies allows for a faster detection and also includes small, slow-proliferating colonies. Scoring errors due to confluence will be avoided using this procedure. The doubling time was determined on the base of CFU-F using the following equation: doubling time  =  ln(2)× culture time [h]/ln((total cell number/((CFU-F/BM-MNC)× cell number seeded)). Cell count was performed using trypan blue to discriminate dead cells.

### Quality Controls of Starting Material, Intermediate and Final Product

Testing of sterility of the starting material (300 µL BM in 20 mL of PBS) as well as of the intermediate and final product (20 mL of cell culture supernatant before harvest enriched with harvested at least 1×10^6^ cells MSC) was performed in accordance with the European Pharmacopeia 2.6.1 using aerobic (BacT/ALERT BPA, bioMérieux SA, Nürtingen, Germany) and anaerobic (BacT/ALERT BPN, BioMérieux, Nürtingen, Germany) culture bottles in the BacT/ALERT 240 system from BioMérieux (Nürtingen, Germany).

Endotoxin testing of culture supernatant at harvest of intermediate and final product was performed according to European Pharmacopeia 2.6.14 by L&S, Labor (Germany, Bad Bocklet-Großenbrach) using a Limulus Amoebocyte Lysate (LAL)-test.

### Karyotyping

Karyotyping was performed by a certified clinical cytogenetics laboratory (Institute for Human Genetics, University of Ulm, Ulm, Germany). For karyotyping, cells of passage 1 were cultured at a density of 30.000 MSC per two T-75 flasks (NUNC, Wiesbaden, Germany).

Briefly, 750 mg/mL colchicin was added to the cell culture at 40% cell confluence for at least 60 minutes to stop cell division at mitosis and allows an increased yield of mitotic cells for analysis. Cells are then transferred in hypotonic solution causing cell swelling and fixed using methanol/acetic acid and transferred to slides for further analysis.

After fotodocumentation, routine analysis of G-banded chromosomes was performed according to standard procedures as described elsewhere [8]. Band resolution was 300–400 per haploid chromosome set. A total of 28±10 mitoses were analyzed (n = 13).

### Flow Cytometric Characterization of MSC

Antibodies used for characterization included CD3 (Clone SK7), CD9 (M-L13), CD34 (8G12), CD40 (5C3), CD45 (2D1), CD49a (SR84), CD49c (C3.11.1), CD49d (9F10), CD49f (GoH3), CD56 (NCAM16.2), CD71 (M-A712), CD73 (AD2), CD90 (5E10), HLA-DR,DP,DQ (Tü39), HLA-ABC (G46-2.6), CD140a (αR1), CD140b (18A2), CD146 (P1H12), CCL5/RANTES (2D5), (all from Becton Dickinson, Heidelberg, Germany), CD105 (SN6) (AbDSerotec, Düsseldorf, Germany), CD49e (NKI-SAM-1), CD194/CCR4 (TG6/CCR4), CD197/CCR7 (TG8/CCR7), CXCR4 (12G5), CD349 (W3C4E11) (all from BioLegend, San Diego, USA), CD271 (ME20.4-1.H4), MSCA-1 (W8B2) (both from Miltenyi Biotech., Bergisch Gladbach, Germany), CD117/c-kit (104D2) (Invitrogen, Darmstadt, Germany), CD191/CCR1 (141-2) (MBL, Nagano, Japan), CD166 (3A6) (Lifespan Biosciences Inc., Seattle, USA), CD31 (WM59), CD51 (RMV-7), CD193/CCR3 (5E8-G9-B4), CD29 (TS2/16) (all from eBioscience, Frankfurt, Germany), CD195/CCR5 (T21/8), CD200 (325516), CXCR7 (358426), CD309/VEGFR (89106), SSEA-4 (MC-813-70) (all from R&D Systems, Wiesbaden-Nordenstadt, Germany). All reagents were used according to manufacturers’ recommendations. Relative fluorescence intensity of cells was then acquired using a FACScan (BD Immunocytometry Systems, Heidelberg, Germany) with CellQuest 3.3 Software and a FACSAria with FACS DIVA 6.2 Software (BD Immunocytometry Systems, Heidelberg, Germany). Combinations of antibodies are shown in **Table S1**.

### Differentiation Assays

#### Histochemistry

2.75×10^3^−10^4^ cells/cm^2^ were seeded and differentiation was induced according to the manufacturers’ instructions (adipogenic differentiation medium from LONZA, Basel. Switzerland), chondrogenic and osteogenic differentiation media from Miltenyi, Bergisch Gladbach, Germany). Following differentiation, cells were fixed in 4% paraformaldehyde and osteogenic differentiation was detected showing alkaline phosphatase activity using the FAST™ BCIP®/NBT kit from Sigma-Adrich (Schnelldorf, Germany) according to the manufacturer’s protocol. For adipogenic differentiation cells were fixed in a 3.7% formaldehyde solution and cells were equilibrated in deionized water (B. Braun, Melsungen, Germany) Staining was performed using a saturated Oil Red O solution in 2-propanol (Sigma, Schnelldorf, Germany). The saturated Oil Red O solution was diluted to 60% using deionized water and filtered to obtain a working solution. Cells were stained for 4–6 min with this working solution after equilibration for 2–5 min in 2-propanol, rinsed in tap water until water was clear and filtered counterstaining with filtered Haematoxylin-Harris solution (Sigma, Schnelldorf, Germany) according to the manufacturer’s instructions.

Chondrogenic differentiation was performed by Methylene Blue staining. Cells were fixed for 8–15 min, air-dried and stained for 80–120 min using a Methylene Blue solution (Merck, Darmstadt, Germany). Stained slides were carefully rinsed two times in deionized water and air-dried.

#### Real-time reverse transcriptase polymerase chain reaction (real-time RT-PCR) and semi-quantitative PCR

Quantitative effects on gene expression were examined as previously described [9].

Briefly, RNA was isolated from triplicate cultures in 24-well plates (osteogenic and adipogenic differentiation) or quadriplicate pellet culture (chondrogenic differentiation) using the RNeasy Mini Kit (Qiagen, Hilden, Germany) according to the manufacturer instructions. 1 µg RNA was transcribed into cDNA using the Omniscript RT Kit (Qiagen, Hilden, Germany). Specific primer pairs for transcription factor Runx2, bone sialo proteine (BSP), alkaline phosphatase (AP), osteopontin (OP), osteocalcin (OC), transcription factor Sox9, aggrecan (Agg) and collagen type 2 (COL type 2) were designed using published gene sequences (PubMed, NCBI Entrez Nucleotide Database, see Tautzenberger et. al. [9]) and synthesised by Thermo Electron Ulm (Ulm, Germany). Amplification products were cloned and used as standards for real-time RT-PCR (StepOnePlus™ Real-Time PCR System, Applied Biosystems, Darmstadt, Germany). The amount of each respective amplification product was determined relative to the house-keeping gene GAPDH. Normalised values of differentiated cells were compared to the control at day 0.

In undifferentiated MSC at day 0 the expression of adipogenic marker genes PPARγ and LPL was not detectable, respectively it was extremely low (at the detection limit). For this reason the quantitative RT-PCR analysis of peroxisome proliferator activated receptor γ (PPARγ) and lipoproteinlipase (LPL) comparing the mRNA expression in differentiated cells at day 21 and in undifferentiated control at day 0 was not possible. To analyse adipogenic differentiation we performed semi-quantitative RT-PCR analysis. cDNA produced as described above was amplified using specific primer pairs for LPL and PPARγ and HotStarTaq DNA polymerase (Qiagen, Hilden, Germany) with a 32 cycle PCR programme (*RoboCycler*® GRADIENT 96, Stratagene, Waldbronn, Germany). The amplification products were separated on a 2% agarose gel (Life Technologies, Darmstadt, Germany), visualised by ethidium bromide staining and documented with a gel documentation system (QUANTUM ST4-3000, Vilber Lourmat, Eberhardzell, Germany).

### Lactate and Glucose Analysis

Lactate concentration in cell culture supernatant was measured using the Lactate Pro Blood Lactate Test Meter (ARKRAY Inc., Amstelveen, The Netherlands) following manufacturer’s instructions. The Lactate Pro Test Strip has a measuring range of 0.8–23.3 mmol/L and this device conforms to the Directive 98/79/EC.

Glucose concentration in cell culture supernatants was analyzed using the Contour Blood Glucose Monitoring System (Bayer Vital GmbH, Leverkusen, Germany) which has a measuring range of 10–600 mg/dL and was used according to manufacturer’s instructions.

### Cytokine Analysis

Custom-designed MILLIPLEX® Human Cytokine/Chemokine 96-well Plate Assays (Cat. #MPXCYTO-60K, #HNDG3-36K, #TGFB-64K-01, Millipore Corporation, Billerica, USA) were used for the simultaneous quantification of human cytokines and chemokines of cell culture supernatants as per the manufacturer’s specifications.

Cell culture supernatant samples were collected before and during cell cultivation at day 3, 7, 10, 13, 14, 17, and 21, i.e. usually before washing, after medium exchange and before harvest. At each time point of interest, an aliquot of the cell culture supernatant was immediately stored at −80°C. All frozen aliquots were subsequently thawed and analyzed simultaneously via the MILLIPLEX Cytokine Assay on a Luminex LS-100 platform. Samples were processed in a way that they did not differ in number of freezing/thawing cycles. Data are expressed as mean ± standard deviation.

### Statistics

Comparison of means was performed using unpaired, two-sided Student’s T-test. Threshold for highly significant difference was p<0.01, for significant difference was p<0.05.

## Results

### Quality Controls

All samples (BM, intermediate product, final product) were negative in BacT/ALERT testing and endotoxin concentration of all samples was less than 1 I.U./mL. Karyotyping was without pathological findings. No clones with chromosomal abnormalities were found by karyotyping in any of the large-scale expansions analyzed.

### Analysis of Starting Material

Five different GMP-compliant large-scale isolation/expansion protocols as described in **Table 1** and illustrated in **Figure 1** were tested.

The age of donors ranged from 18 to 37 years (**Table S2**) and the transportation time between BM collection and seeding of BM to the CellSTACKS was prolonged for BM acquired by purchase as compared to freshly collected BM. However, there was no significant difference in donor age for single-step versus the four different two-step systems (p≥0.2) and no significant difference in donor age (p≥0.4) and transportation time (p>0.5) between the different two-step systems.

**Figure 2:**
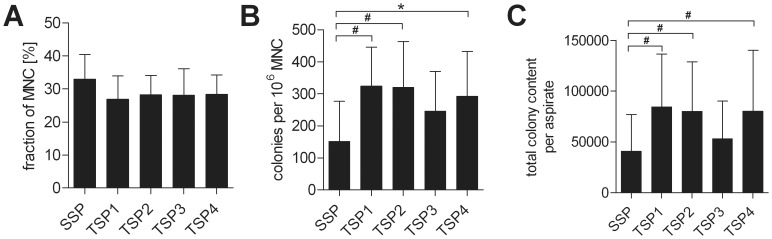
Analysis of starting material. **A**. Fraction of MNC. **B**. Colonies per million MNC C. Total colony content per BM aspirate or the starting material (unprocessed bone marrow) used for expansion according to the single-step (SSP) or the two-step protocols (TSP1 - 4). *indicates statistically highly significant (p<0.01). #indicates statistically significant (p<0.05).

The amount of colony-forming units fibroblasts (CFU-F) per million BM-MNC differed significantly between the single-step protocol and protocol TSP1 (p<0.05), TSP2 (p<0.05) and TSP4 (p<0.01) of the two-step protocols. However, the total number of CFU-F in the BM aspirates used in the four two-step protocols protocols did not differ significantly (p>0.2) (**Figure 2**).

### Analysis of MSC Yield, Population Doublings and Clonogenicity

**Figure 3:**
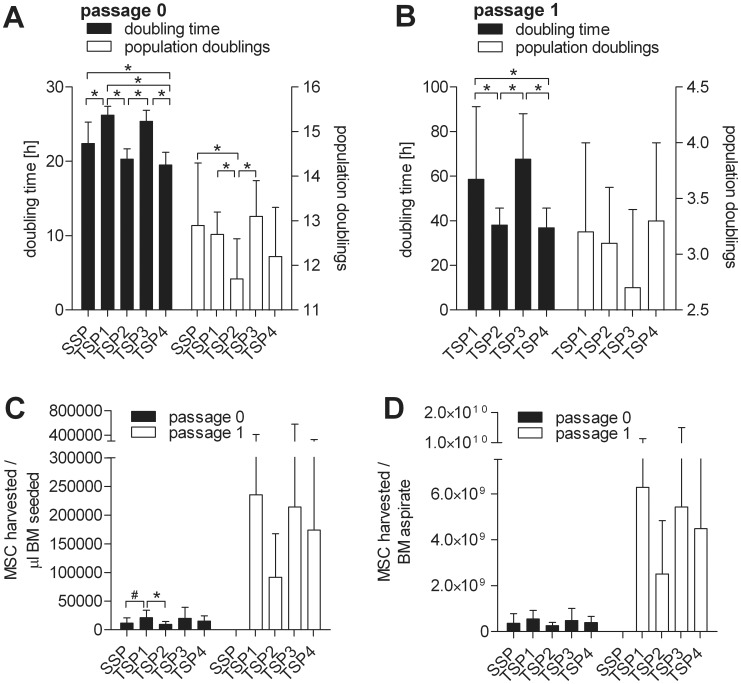
Comparative analysis of MSC yields. Shown are mean and SD values of A. doubling time and populations doublings of passage 0, B. doubling time and populations doublings of passage 1, C. MSCs harvested per ml BM seeded and D. MSCs harvested per total BM aspirate for all of the established GMP-compliant expansion protocols at passage 0 and passage 1. *indicates statistically highly significant (p<0.01). #indicates statistically significant (p<0.05).

As summarized in **Figure 3** **and Table S3**, 49.3×10^3^ to 50.6×10^3^ BM-WBC (corresponding 9.6×10^3^ to 19.4×10^3^ BM-MNC) per cm^2^ were seeded in two-step protocols whereas seeding density was 22.9×10^3^ to 62.3×10^3^ BM-WBC (corresponding 11.8×10^3^ to 12.2×10^3^ BM-MNC).

MSC of the single-step system were harvested after 12.9±1.4 population doublings (12.0±1.5 days) with a doubling time of 22.4±2.9 hours at a density of 14.2×10^3^±11.6×10^3^ MSC/cm^2^ and a viability of 93.9±8.3% (**Table S3**).

Passage 0 (p0) cells of two-step system protocol option 1 (TSP1) and TSP3 were harvested after 13.8±0.1 days (12.7±0.5 population doublings for TSP1; 13.1±0.8 population doublings for TSP3) with doubling times of 26.2±1.2 and 25.4±1.5 hours at densities of 28.2×10^3^±14.3×10^3^ and 29.3×10^3^±21.6×10^3^ MSC/cm^2^ and viabilities of 96.2±3.9% and 92.4±7.4%, respectively (**Table S3**).

Passage 0 cells of two-step system TSP2 and TSP4 were harvested after 9.9±0.1 days (11.7±0.9 population doublings for TSP2; 12.2±1.1 population doublings for TSP4) with doubling times of 20.3±1.4 and 19.5±1.7 hours at densities of 14.7×10^3^±6.9×10^3^ and 20.8×10^3^±14.0×10^3^ MSC/cm^2^ and viabilities of 95.9±4.1% and 94.4±8.5%, respectively (**Table S3**).

Passage 1 (p1) cells of two-step system protocol option 1 (TSP1) and TSP3 were harvested after additional 6.9±0.3 and 7.0±0.4 days (3.2±0.8 population doublings for TSP1; 2.7±0.7 population doublings for TSP3) with doubling times of 58.5±32.7 and 67.6±20.4 hours at densities of 41.5×10^3^±17.3×10^3^ and 28.6×10^3^±18.0×10^3^ MSC/cm^2^ and viabilities of 95.8±3.4% and 95.0±3.8%, respectively (**Table S4**).

Passage 1 cells of two-step system TSP2 and TSP4 were harvested after additional 4.8±0.3 and 4.9±0.5 days (3.1±0.5 population doublings for TSP2; 3.3±0.7 population doublings for TSP4) with doubling times of 38.1±7.6 and 36.9±8.8 hours at densities of 36.9×10^3^±14.1×10^3^ and 44.4×10^3^±17.9×10^3^ MSC/cm^2^ and viabilities of 94.8±5.9% and 96.0±2.1%, respectively (**Table S4**).

There was a highly significant difference (p<0.01) in doubling times of p0 and p1 for all protocol options of the two-step system. Doubling times differed between the single-step system and TSP2 (p = 0.018) and TSP3 (p = 0.027) of p0 in the two-step system, however, not highly significant. Interestingly, the doubling times in passage 0 between the different two-step systems were significantly different for TSP1 and TSP2 (p<0.01), TSP1 and TSP4 (p<0.01), TSP2 and TSP3 (p<0.01), and TSP3 and TSP4 (p<0.01).

Based on these data, the ratio MSC/µL BM-aspirate (referred as yield) was calculated for MSC of p0 for all expansion systems, as well as for MSC of p1 for all two-step systems (**Table S5**):

In the single-step system, 11.9×10^3^±9.1×10^3^ MSC were harvested per µL BM-aspirate seeded.

In the two-step systems having a total expansion time of approximately 21 days, the yield in p0 was 21.2×10^3^±12.9×10^3^ MSC per µL BM-aspirate for TSP1 and 19.8×10^3^±19.6×10^3^ MSC per µL BM-aspirate for TSP3; for p1, the yield was 235.3×10^3^±175.4×10^3^ MSC per µL BM-aspirate for TSP1 and 214.3×10^3^±360.6×10^3^ MSC per µL BM-aspirate for TSP3.

For TSP2 and TSP4 with an overall expansion time of approximately 15 days, the yield in p0 was 9.6×10^3^±4.7×10^3^ MSC per µL BM-aspirate and 91.5×10^3^±76.1×10^3^ MSC per µL BM-aspirate in p1 for TSP2 and 15.2×10^3^±9.1×10^3^ MSC per µL BM-aspirate and 173.8×10^3^±151.9×10^3^ MSC per µL BM-aspirate in p1 for TSP4.

Based on the total aspiration volumes (**Table S2**) of 31.1±13.6 mL for the single-step protocol, 25.3±5.3 mL for TSP1, 27.2±8.0 mL for TSP2, 23.2±4.8 for TSP3 and 25.0±7.2 mL for TSP4, the total potential yield was projected for p0 and p1, respectively (**see Table S5**).

A highly significant difference in the yield (MSC harvested/µl BM-aspirate seeded) only could be seen between TSP1 and TSP2 (p<0.01) of the two-step expansion system in p0. Interestingly, no significant difference in the yield of single-step system and any of the two-step-systems could be seen for p0 (p>0.05) with the exception of SSP vs. TSP1 (p = 0.038). The only highly significant difference in yield between p0 of the single-step system and p1 of the two-step systems could be observed for TSP1 (p<0.01), TSP2 (p<0.01) and TSP4 (p<0.01), but not for TSP3 (p<0.05).

### Phenotypic Characterization of MSC by Flow Cytometry

**Figure 4:**
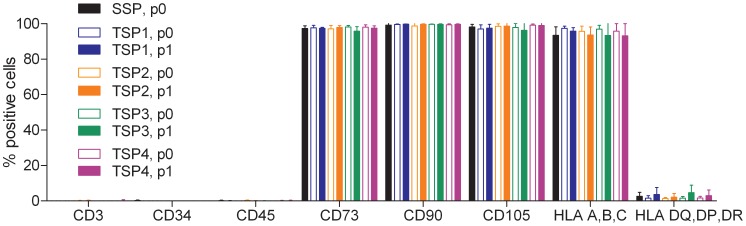
Flow cytometric analysis of MSC isolated and expanded according to a GMP-grade single-step or two-step protocol options. Results of single-step MSC passage 0 (n = 15), two-step protocol 1 (TSP1), p0 (n = 8), p1 (n = 8), protocol 2 (TSP2), p0 (n = 10), p1 (n = 10), protocol 3 (TSP3), p0 (n = 6), p1 (n = 6), protocol 4 (TSP4), p0 (n = 11), p1 (n = 12) are shown as mean and SD values.

We have analyzed the expression of surface marker antigens characteristic for MSC as defined by the ISCT [1]. As illustrated in **Figure 4** we found expression on >95% of CD73, CD90, CD105 and HLA-ABC and <1% expression of CD3, CD34 and CD45 of MSC derived from either the single-step or the two-step protocols. HLA-DP, DQ, DR was expressed at a range of 1.3±0.6% –4.7±4.2% on the cell surface. Passaging of MSC expanded according to the two-step protocol options *in vitro* did not significantly influence expression profile of these markers.

Moreover, we also investigated additional surface antigens related to chemotaxis, adhesion, metabolism as well as putative MSC-specific markers to better characterize MSC that were expanded according to either the single-step or any of the two-step protocol.

**Figure 5:**
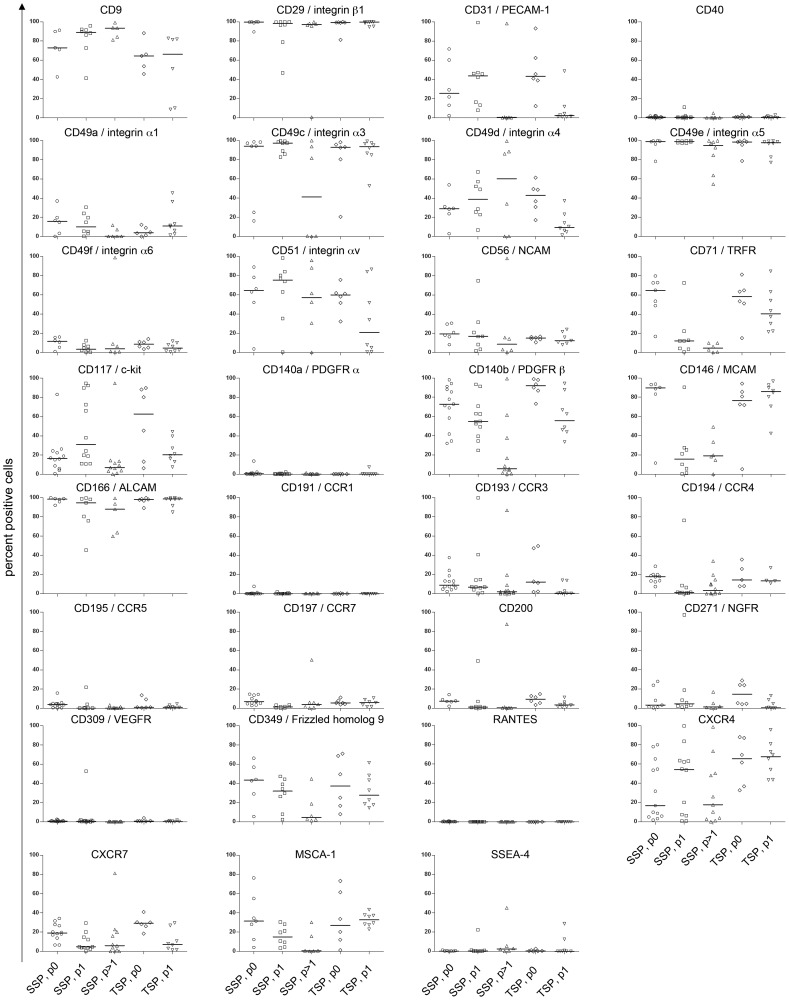
Flow cytometric analysis of MSC expanded according to a GMP-grade single-step or two-step protocol. Results of single-step MSC passage 0 (n = 6–13), p1 (n = 7–12) and p>1 (n = 6–12) as well as two-step MSC at p0 (n = 6) and p1 (n = 8) are shown as percent positive in Tukey’s Whisker Plots.

Interestingly, we could observe subtle differences in median expression pattern of MSC derived from either system as illustrated in **Figure 5**. Whereas integrin β1/CD29 and integrin α5/CD49e were expressed uniformly at median levels ≥98%, and integrin α1/CD49a, integrin α6/CD49f were expressed on less than 12% of the population, integrin α4/CD49d and integrin αν/CD51 showed deviant expression at p1 or higher. While expression of integrin α4/CD49d on the surface of MSC derived from the single-step system increased from a median of 29% at the end of p0 to 37% at p1 and 61% at passages higher than 1 (p>1), median values markedly decreased from 43% at p0 to 10% at p1 on cells cultivated using the two-step protocol. Integrin α ν/CD51 expression was found to be expressed consistently at high levels (65% at p0, 79% at p1 and 57% for p>1) on single-step-derived MSC, but sharply decreased in expression on two-step MSC from 60% at p0 to 21% at p1. Interestingly, integrin α3/CD49c expression sharply decreased from ≥93% at p0 and p1from either system, but then declined to 41% of cells at p>1, indicating that prolonged *in vitro* cultivation may affect expression of this molecule. In addition to adhesion molecules such as integrins, chemokine receptors have been proposed as mechanistic regulators in MSC migration and homing to sites of inflammation [10]. Therefore, we analyzed the chemokine receptor (CCR) profile of MSC and found absence of expression of CCR1/CD191 (median expression ≤0.1%) and an approximately uniform expression of CCR5/CD195 (median expression ≤4%) and CCR7/CD197 (median expression ≤7%) on all MSC analyzed. Comparably, median expression of CCR3/CD193 (9% at p0, single-step; and 12% at p0, two-step), CCR4/CD194 (18% at p0, single-step; 14% at p0, two-step) and CXCR7 (19% at p0, single-step; 29% at p0, two-step) was higher at early passages, but then progressively decreased with cell passaging *in vitro*. In contrast, CXCR4 levels changed considerably on cells derived from the single-step protocol. CXCR4 was detected on 17% of cells at p0, which then increased to 59% at p1 and declined to 18% of cells at p>1, while MSC cultivated via the two-step protocol expressed this marker at 66% at p0 and 68% at p1, respectively.

In a similar fashion, median surface expression levels of CD146 strongly decreased from 90% at p0 to 16% at p1 and 19% at p>1 on single-step-derived MSC, while it was expressed stably at 77% on p0 and at 86% at p1 on two-step-derived MSC.

We also analyzed surface antigens indicative of cell proliferation and differentiation. We observed absence of VEGFR/CD309 and RANTES and low expression levels of PDGF receptor α/CD140a (≤0.4%) on all MSC analyzed. CD40 was also not detectable any of the populations, confirming the absence of hematopoietic cells of the immune system. In contrast to the low levels of CD140a, we found PDGF receptor β/CD140b on 73% of cells analyzed at p0 of single-step MSC and on a median of 92% of two-step-MSC at p0. However, CD140b was then down-modulated *in vitro*, declining to 55% at p1 and 6% at p>1 (single-step) and to 56% at p1 (two-step), respectively. This expression pattern was also detected for transferrin receptor/CD71, which markedly decreased from 65% at p0 to 12% at p1 and 5% at p>1 (single-step) and 58% at p0 to 40% at p1 (two-step) during cell culture. Also following this profile, CD31 strongly decreased to levels ≤0.5% at p>1 (single-step) and ≤1% at p1 (two-step) following cell passaging *in vitro*.

Moreover, we also investigated the expression profile of putative MSC-specific surface antigens suggested for prospective MSC isolation from *in vivo* sources. We found a high expression (median expression ≥64%) of CD9, as well as CD166 (on a median of ≥74%) of all MSC derived from any cultivation system. Conversely, CD56 (median expression ≤20%) and CD200 (median expression ≤9%) were expressed consistently at low levels and the latter subsequently declined to ≤1% median expression at p>1 (single-step) and ≤4% at p1 (two-step). This expression pattern could also be observed for NGFR/CD271, which declined during in vitro cultivation from 3% at p0 to 1% at p>1 (single-step) and from 15% at p0 to 0.5% at p1 (two-step), respectively. Expression of Frizzled homolog 9/CD349 was also markedly down-regulated on MSC derived from either the single-step (median expression of 44% at p0, 28% at p1 and 5% at p>1) or the two-step (median expression of 37% at p0 and 28% at p1) protocols, as did expression of c-kit/CD117 (median expression of 16% at p0, 29% at p1 and 7% at p>1, single-step protocol and 63% at p0, 20% at p1, two-step protocol) further indicating the modulatory effects of prolonged *ex vivo* culture and passaging on MSC phenotype. Interestingly, MSCA-1 was found to be down-modulated on the surface of MSC population derived from the single-step protocol during in vitro propagation (31% at p0, 15% at p1, 0.7% at p>1), but was subtly increased on MSC expanded according to the two-step system (30% at p0, 33% at p1). Finally, expression of SSEA-4 was nearly absent, being expressed on 0.3% of the MSC population at p0, on 0.4% at p1, on 3% at p>1 (single-step) as well as on 0.4% at p0 and on 0.1% of cells at p1 (two-step).

We also analyzed the median fluorescence intensities of MSC positive for their respective surface antigen (**Figure S1**). Most markers present on the surface of MSC uniformly showed stable median expression intensities, regardless of their expansion system. As an exception to this, fluorescence intensities of CD31, CD117, CD271, CD349 and SSEA-4 were stable at p0 and p1 of either protocol, but slightly increased on MSC derived from single-step expansions at higher passages. Notably, these markers were detected only at very low levels on the surface of MSC at higher passages. Therefore, these values may contain numerical artefacts due to the threshold settings used for the calculation.

### In vitro Differentiation Capacity

**Figure 6:**
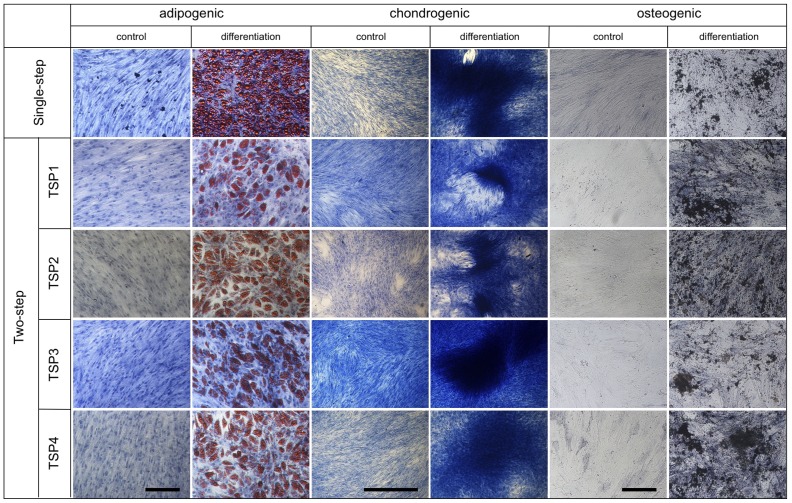
Differentiation capacity of representative MSC batches isolated and expanded using the five different cell expansion systems. Adipogenic (Oil RedO/haematoxylin staining), chondrogenic (methylene blue staining) and osteogenic (detection of alkaline phosphatase) differentiation assays are shown. Control assays were performed in aMEM supplemented with 10% FCS. Black bar indicates 1000 µm for adipogenic and 100 µm for chondrogenic and osteogenic differentiation.

Histochemical analysis of MSC differentiation for all five isolation/expansion systems is shown in **Figure 6**. Differentiation capacity of preparations of all five different isolation/expansion systems was similar.

The mRNA analysis show increased expression of osteogenic and chondrogenic differentiation markers in most of MSC populations when compared to non-differentiated control at day 0 (**Figure S2 and S3**). A slight up-regulation of Runx2 was found in MSC derived from 7 in presence of FCS (donor 5-FCS). Collagen type 2 mRNA could be measured at day 30 in MSC pellet culture under chondrogenic conditions but not at day 0 (**Figure S3**). In MSC isolated in medium supplemented with FCS (n = 3) the up-regulation of AP was significant higher (p<0.005) when compared to MSC isolated in presence of PL (n = 5). Regarding adipogenic differentiation a clear up-regulation of LPL, as well as of PPARγ was found in all MSC populations. The representative results obtained with donor 3 are presented in **Figure S4**.

### Analysis of Metabolic Parameters and Cytokine Profiles in the Medium

**Figure 7:**
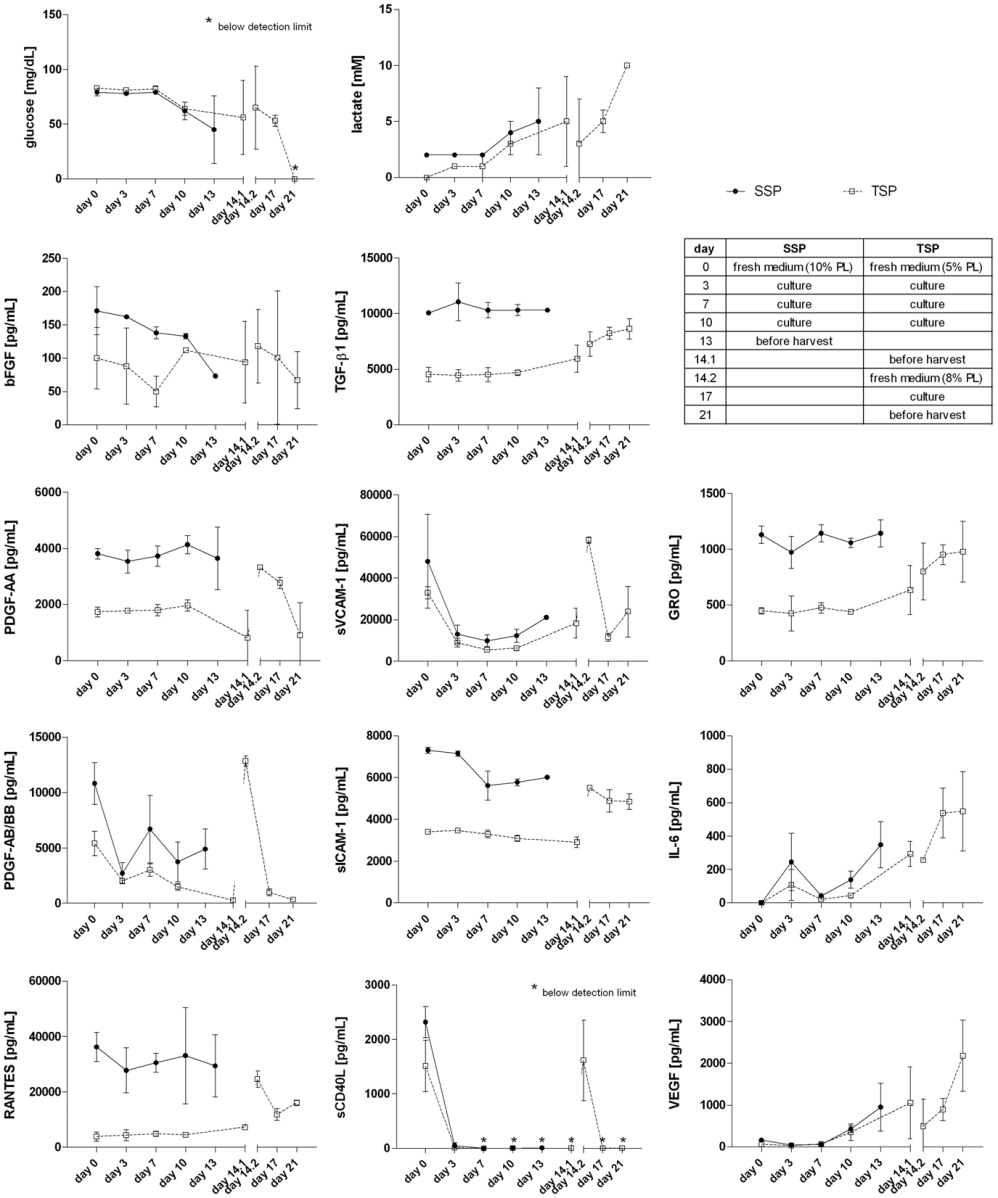
Quantitative cytokine analysis and acquisition of metabolic parameters of single-step and two-step MSC expansion media. Samples of cell culture supernatants were collected at the time points indicated during single-step and two-step (*TSP1*; 14+7-5-8) GMP-grade large-scale cell expansion runs and analyzed for their cytokine content. Depicted are results of glucose, lactate, bFGF, IL-6, GRO (CXCL1/2/3), sCD40L, RANTES/CCL5, PDGF-AA, VEGF (n = 4 single step runs; n = 3 two-step runs), sVCAM-1, PDGF-AB/BB (n = 3 single step runs; n = 3 two-step runs), sICAM-1, and TGF-β1 (n = 2 single step runs; n = 3 two-step runs) contents as mean and SD values.

We also wanted to investigate the cytokine release and consumption profile as well as the metabolic state of MSC during *in vitro* expansion. To this end, we analysed cell culture supernatants of cells that were expanded according to the single-step protocol and compared results with those derived from the two-step procedure TSP1 (**Figure 7**). Samples were collected after cell seeding in fresh medium containing 5, 8 or 10% PL as supplement, during cell expansion and before harvest and reflect the sum of soluble factors secreted and absorbed by MSC *in vitro*.

**Table 2:** Comparison of the different protocols and their outcomes.

System	single-step	two-step			
Protocol	SSP	TSP1	TSP2	TSP3	TSP4
Units needed to obtain100×10^6^ MSC*	3×5-chamberstack	p0: 1×2-chamber stack p1: 2×2-chamber stack	p0: 1×2-chamber stack p1: 3×2-chamber stack	p0: 1×2-chamber stack p1: 3×2-chamber stack	p0: 1×2-chamber stack p1: 2×2-chamber stack
Volume of BM needed toseed this number of stacksin p0	12.7 mL	1.9 mL	2.2 mL	2.2 mL	2.0 mL
Volume of mediumneeded to seed thisnumber of stacks	6.0 L	2.1 L	1.5 L	2.1 L	1.5 L
Volume of PL needed toseed this number of stacks	600 mL	132 mL	93 mL	210 mL	150 mL
Overall expansion time	12.0 days	20.7 days	14.7 days	20.8 days	14.8 days
Population doublingsper day	1.08	1.05	1.01	0.76	0.77
Trilineage-differentiationcapacity	passed	passed	passed	passed	passed
Surface marker expressionof standard panel	passed	passed	passed	passed	passed
Surface marker expressionof extended panel;at p0 to p>1	increasing expressionof integrins α4 and αv, loss of CD46 and MSCA-1	loss of expression of integrins α4 and αv, stable expression of CD146 and MSCA-1			

* always rounded up to next higher integer. All values represent mean values.

As shown in **Figure 7**, the overall cytokine profile of MSC derived from a single-step protocol did not differ significantly compared to levels found in culture supernatants of MSC from a two-step protocol system. We observed a progressive decline of sICAM-1 levels as well as a sharp decrease in sCD40L levels which dropped below detection limit on day 3 after cell seeding in fresh medium. Analysis of bFGF, PDGF-AA and PDGF-AB/BB levels also revealed an overall decrease during *ex vivo* cell culture, whereby higher levels could be detected in supernatants after exchange of cell culture media. Likewise, levels of RANTES declined after cell seeding, and could then be quantified as a variable, but overall stable continuum and even showing a slight increase towards the end of culture at p0 and p1 of the two-step cell expansion system. Similarly, quantification of TGF-β1 showed little variation in media samples of MSC cultivated in the single-step system, but was found to increase slightly at the end of p0 and in p1 in culture medium of MSC expanded according to the two-step protocol. This pattern was also observed regarding GRO (CXCL1/2/3) levels, which were found to increase, albeit showing variable quantities in p1 of the two-step system. Interestingly, analysis of sVCAM-1 revealed a sharp decline in levels at day 3 compared to those in fresh media, which then, however, again increased progressively until cell harvest, suggesting that this factor may also be secreted by MSC. Finally, levels of VEGF and IL-6 markedly ascended during MSC expansion, being at or below detection limits after initiation of cell culture and subsequently rising to 348±138 pg/mL (single-step, p0) or 548±238 pg/mL (two-step, p1) and 950±567 pg/mL (single-step, p0) or 2178±852 pg/mL (two-step, p1) before harvest of the final product, respectively.

Furthermore, we then quantified glucose consumption and lactate production by MSC which were cultivated via the single-step or the two-step system to assess the metabolic state *in vitro* during cell expansion. The results (**Figure 7**) reveal mostly stable levels of glucose and lactate during the first 7 days of culture of MSC in p0. However, glucose levels then declined progressively, also showing marked variation at the day of cell harvest. Conversely, lactate content was found to increase in this period. Notably, cell culture supernatants of MSC expanded in the two-step system then showed a strong decrease in glucose levels in p1, and subsequently dropped below detection limit at the day of harvest. This process was mirrored by levels of lactate which ascend to 10±0 mM at day 21 (p1). Collectively, these results comprehensively document and monitor the dynamic cytokine continuum of proliferating MSC during *ex vivo* expansion in two GMP-grade clinical-scale cultivation systems.

## Discussion

Apart from the source of isolation, the manipulation of the source material as well as tissue culture components and conditions, e.g. oxygen tension, temperature, basal media and additives may alter the cell phenotype and its functions. Cell expansion protocols defining cell-seeding density, media formulation and exchange during *in vitro* cultivation are still laboratory-specific and not standardized in any of these given factors.

Interpretation of experimental data therefore contains some caveats and requires knowledge of cell isolation technique, media composition and cell doublings to make an informed choice regarding the *in vitro* cell cultivation for clinical application.

In the United States, MSC are considered in the context of human cells, tissues, or cellular and tissue-based products (HCT/Ps). Therefore, MSC production must be in compliance with The Code of Federal Regulation (CFR), Title 21, Part 1271 [11] and in accordance with current Good Tissue Practice (cGTP) requirements as described in ‘Current Good Tissue Practice (CGTP) and Additional Requirements for Manufacturers of Human Cells, Tissues, and Cellular and Tissue-Based Products (HCT/Ps)’ [12].

In Europe, MSC are considered as advanced therapy medicinal products (ATMPs), as defined by the European Regulation EC 1394/2007. [5]. Depending on the source, manufacturing process and intended application, MSC may be considered somatic-cell therapy products or tissue-engineered products [13]. The European Regulation EC 1394/2007 refers to the European GMP guidelines and is in compliance with the 2003/94/EC directive on medicinal products for human use [14] as well as directive 2002/98/EC [15] setting standards of quality and safety for the collection, testing, processing, storage and distribution of human blood and blood components.

Hence, regulatory authorities mandate cell production according to GMP-grade system with appropriate and rigorous process validation and quality controls. Quality controls must include bacteriological tests, phenotypic control, viability, safety and efficacy of the final product. Release criteria for the final product must be defined and met by each therapeutic product.

To date, published data does not infer a propensity of human MSC to develop morphological or genetic alterations during *ex vivo* propagation. In particular, a recent report shows that MSC derived from bone marrow or umbilical cord blood and expanded in the presence of either PL or FCS could be cultivated up to passage 25 or senescence without loosing their phenotypical and functional characteristics [16]. Other data shows occurrence of aneuploidy in MSC during *in vitro* cultivation, yet this did not result in cell transformation, but rather in cellular senescence [17]. Clinical prudence and regulatory agencies do, however, require preclinical safety data of the therapeutic product as a part of quality control [5], [13]. Hence, we examined the karyotype of MSC isolated and expanded according to the different GMP-compliant protocols to investigate potential genetic instability. No clones with chromosomal abnormalities were found by karyotyping in any of the large-scale expansions analyzed.

The surface antigen profile of MSC is characterized by absence of hematopoietic cell markers, such as CD3 (T cell receptor), CD34 (hematopoietic progenitor cell antigen 1) and CD45 (common leukocyte antigen) and strong expression of CD73 (ecto-5′-nucleotidase), CD90 (Thy-1) and CD105 (endoglin). These minimal criteria were defined by the ISCT [1] to augment standardization of cell preparations and set a basis for harmonized and comparative cell expansions among laboratories. Additional characterization of this cell population is firmly encouraged and required to further delineate the effect of *ex vivo* conditions and expansion settings on MSC biology.

We analyzed the minimal criteria for all five expansion systems and investigated the presence of further informative surface antigens via flow cytometry, yet found that a singular and precise phenotypic definition of MSC to be hampered by the heterogeneity of the cultivated cell population(s).

While we were able to confirm the presence of known quality-control markers such as CD29, CD73, CD90, CD105, CD166 as well as the absence of CD3, CD34, CD45 and HLA-DR, we also detected marked variability in a great spectrum of expression markers. We also investigated the presence of putative MSC-characteristic unique markers for suggested for the prospective isolation from bone marrow or other tissues, e.g. CD9, CD56, CD200, CD271, CD349, MSCA-1 and SSEA-4 [18], [19] and found expression of CD200, CD271, SSEA-4 on ≤5% of MSC at the passage for clinical application e.g. passage 0 following the single-step protocol and passage 1 following the two-step protocol. Interestingly, we found considerable expression of CD349 and MSCA-1 on MSC in early passages, suggesting that a subset of MSC is indeed present in the heterogeneous *in vitro* cell population as described [19], [20]. Finally, while CD9 was consistently found to be expressed by MSC (median expression ≥64%), expression of CD56 was detected only on ≤20% of the cell population.

Furthermore, we could show the impact of prolonged *in vitro* cultivation on the MSC antigen profile, resulting in the down-regulation of surface markers integrin α6, integrin αν, CD71, CD140b, CCR4, CD200, CD271, CD349 and CXCR7 with passaging.

Recent work has shown that MSC are capable of migrating towards injured tissues, such as myocardium, lung, pancreas, skin and bone and play an integral role in modulating the local microenvironment to enhance tissue regeneration [4]. Our comprehensive analysis of surface antigens shows that MSC express chemokine receptors CCR3, CCR4, CCR5, CCR7, CXCR4 and CXCR7 as well as integrins α1, α3, α4, α5, α6, αν and β1, suggesting that these cells are capable of sensing and being recruited to sites of inflammation, possibly following the multistep cell homing model described by Henschler *et al*. [10].

In summary, passaging of cells *in vitro* resulted in distinct changes of their surface receptor profile, further illustrating the functional complexity and the internal heterogeneity of cells expressing the characteristic ISCT-compliant standard MSC markers. Therefore, the conceptual simplification of the term “MSC" is debatable, as a clear phenotypic definition of MSC is hampered by the non-homogeneous surface marker expression profile of the cells affected by the local microenvironment. However, whether these phenotypical differences observed *in vitro* will subsequently be of clinical relevance remains to be elucidated.

The hallmark biologic property of MSC is their capacity for trilineage mesenchymal differentiation into osteoblasts, adipocytes and chondroblasts using standard *in vitro* tissue culture-differentiating conditions. We verified and documented this unique property via histochemical stainings and quantitative real-time PCR for all MSC isolated and expanded according to any of the five protocols presented.

No obvious difference in histochemical staining for adipogenic, chondrogenic and osteogenic differentiation was detectable among the five different expansion protocols. However, quantification of data from histochemical staining is difficult and bears the error of subjective differentiation. This potential error was overcome by performing qPCR analysis of differentiated MSC.

The results of mRNA analysis confirmed the histochemical evaluation. The expression of osteogenic, chondrogenic and adipogenic marker genes could be found in MSC isolated in medium containing PL, as well as in MSC isolated in presence of FCS. Considerable donor variability in mRNA expression of all marker genes was observed in both populations before and after osteogenic and chondrogenic differentiation. This could be a result of cellular heterogeneity among the donor populations as previously reported [21], [22].

Effect of PL on MSC differentiation is disputed. Stimulation of strong proliferation but inhibition of differentiation by PL has been found in rat and human MSC [23], [24]. Increase in osteogenic differentiation and decrease in adipogenic differentiation of human MSC in presence of PL was reported [25]. The results of present study evidence osteogenic, chondrogenic and adipogenic differentiation capacity of MSC isolated in presence of FCS or in xenogen-free medium containing PL and confirmes published results [26].

The cytokine milieu present may critically determine cell behaviour and affect tissue regeneration, as alterations in the expression of homing and adhesion molecules as well as chemokines and their receptors may favour MSC-mediated immune-modulation and ameliorate pathological environments, facilitate efficient engraftment and support wound healing. Published data shows that MSC are able to secrete a variety of bio-active molecules, such as interleukin (IL)-6, IL-7, IL-8, IL-12, IL-14, IL-15, macrophage-stimulating factor (M-CSF), granulocyte-colony-stimulating factor (G-CSF), chemokine ligands CCL2, CCL4, CCL5, CCL20, CX3CL1, CXCL8 and CXCL12/SDF-1a [27] as well as bFGF, VEGF, TGF-β1, GRO and RANTES [28], [29], [30], [31]. In addition, the medium supplement platelet lysate (PL) also contains a multitude of cytokines, such as IL-6, VEGF, sCD40L, sVCAM-1, sICAM-1, PDGF-AA, PDGF-AB/BB, RANTES/CCL5, GRO, TGF-β1, and bFGF [6]. Herein, we have comprehensively analysed the cytokine content of cell culture supernatants at different time points during cell cultivation and found comparable cytokine profiles of MSC expanded via the single-step or the two-step procedure. Our results show an increase of IL-6, VEGF, TGF-β1, GRO (CXCL1/2/3), RANTES, and sVCAM-1 levels during or towards the end of *ex vivo* cell expansion, consolidating previous findings [29], [30], [31] and suggesting novel components of the MSC secretome. Interestingly, whereas sVCAM-1 content was found to increase after a strong initial decline in the first three days of cell culture compared to values detected in fresh media, sICAM-1 levels exhibited a gradual decrease during cell cultivation. However, this is in accordance with a previous report by Hwang *et al*. who could detect sICAM-1 expression by amnion and decidua-derived MSC, but not by BM-derived MSC [28].

The sharp decrease of sCD40L on day 3 after cell seeding confirmed our previous quantification of this factor in PL, which was found to diminish rapidly if PL was stored at 37°C [6]. Likewise, levels of well-established growth stimulating factors PDGF-AA, PDGF-AB/BB and bFGF declined progressively in cell culture media, but could be replenished to some extent by media exchange. The observed variations in cytokine contents as quantified in cell culture supernatants reflect donor-dependent inter-individual variability as well as heterogeneity of MSC populations. In addition, we found that glucose consumption and lactate production to be pertinent surrogate parameters to conveniently assess metabolic state of proliferating cells. While glucose concentration progressively declined during cell expansion, lactate levels ascended, reaching maximum values at the day of harvest. Again, the continuous pattern of both markers showed high similarity when comparing the single-step and the two-step MSC expansion systems.

In sum, these data provide a system-wide comprehensive analysis of the *in vitro* cytokine continuum during clinical-scale cell expansion and complement the complexity of MSC surface receptome. Moreover, our results further suggest that subtle differences in micro-environmental contexts may collectively govern MSC population dynamics and will aid in the interpretation of secretory and migratory cell behaviour. As these quantified bioactive molecules affect the immediate cellular milieu and imply close juxta-positioning with communicating cells, it may be assumed that a deeper mining of this and additional data sets could also reveal yet-unidentified immunomodulatory mechanisms exerted by MSC cross-talk with other cells of the immune system in inflammatory settings.

### Conclusion

Data presented here unequivocally show that the five different systems for isolation and expansion of MSC described in this study are suitable to produce at least 100 millions of MSC, which is commonly regarded as a single clinical dose [3].

In addition to a single-step procedure, this study presents four two-step protocol options. As summarized in **Table 2**, there are certain advantages of some expansion protocols. The single step protocol, e.g., may be suitable for time sensitive expansion purposes, as within 12 days, a target of 100 millions of MSC can be achieved. In contrast, the single step system causes higher costs for material, medium and supplement and also is more labour-intensive.

Except for the different expansion times of 14 and 7 days (TSP1 and TSP3) versus 10+5 days (TSP2 and TSP4), the advantage between the different protocol options of the two-step systems is marginal. Astonishingly, the use of a higher concentration of PL in TSP3 and TSP4 does not have an effect on the number of population doublings per day. This factor, however, is influenced by the culture time.

The shorter protocol options running 10 and 5 d, respectively, are more favourable in terms of economy by saving labour and reagents necessary for the 14 and 7 days of expansion. The shorter protocol options may sometimes fail to produce the required cell numbers within the short time frame. The four two-step protocols offer flexibility in the expansion modality and can be selected with respect to the patient and necessary cell number for clinical application.

If less than 100 millions of MSC are needed, both the single-step and the shorter options of the two-step protocols may be more suitable.

Final products are identical according to the minimal criteria for MSC defined by the ISCT. Moreover, we could show that chemokine and integrin receptors analyzed showed the same expression pattern, suggesting that MSC from either of the isolation/expansion-systems show identical characteristics of homing and adhesion.

## Supporting Information

Figure S1 **Flow cytometric analysis of MSC cultivated using a GMP-grade single-step or two-step protocol.**
(DOCX)

Figure S2 **Expression of osteogenic markers in MSC during differentiation.**
(DOCX)

Figure S3 **Expression of chondrogenic markers in MSC during differentiation.**
(DOCX)

Figure S4 **Expression of adipogenic markers in MSC derived from donor 3 during differentiation upon adipogenic conditions.**
(DOCX)

Table S1 **Combination of markers used for flow cytometric analysis of MSC cultivated using a GMP-grade single-step or two-step protocol.**
(DOCX)

Table S2 **Characterization of starting material for single-step and two-step cell expansion protocols.**
(DOCX)

Table S3 **Summary of results for passage 0 for the different expansion protocols.**
(DOCX)

Table S4 **Summary of results for passage 1 for the different two-step expansion protocol options.**
(DOCX)

Table S5 **Calculations on the potential MSC-yield for the different expansion systems.**
(DOCX)
