# Supplementary material for: GMP-Compliant Isolation and Large-Scale Expansion of Bone Marrow-Derived MSC
Source: PLoS One. 2012 Aug 14;7(8):e43255. doi: 10.1371/journal.pone.0043255 (PMC3419200; doi:10.1371/journal.pone.0043255)
Supplement: Figure S1 — Flow cytometric analysis of MSC cultivated using a GMP-grade single-step or two-step protocol. (DOCX) [file pone.0043255.s001.docx]

**Supplementary Figure S1:** **Flow cytometric analysis of MSC cultivated using a GMP-grade single-step or two-step protocol.**


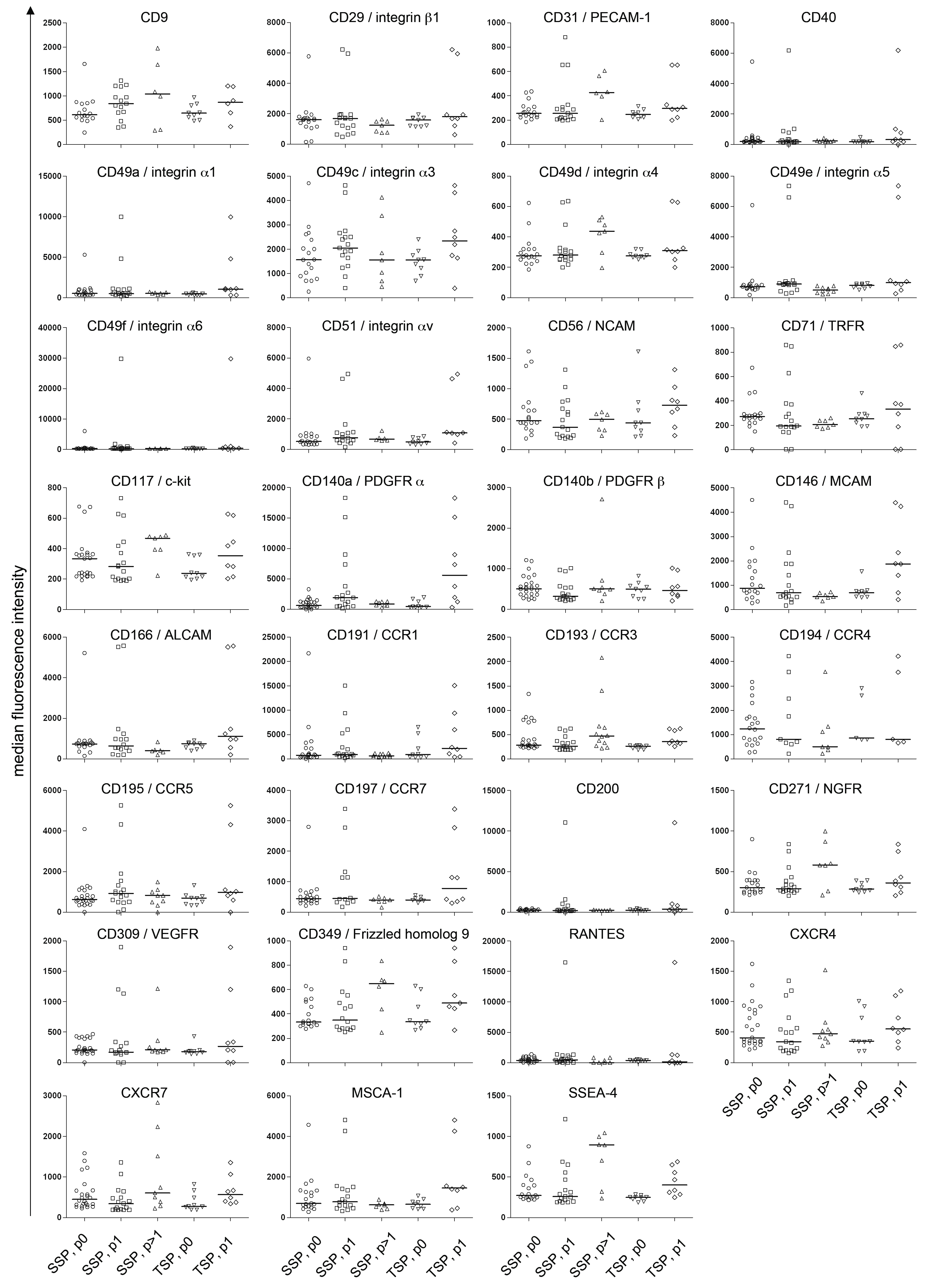


Median Fluorescence Intensities (MFI) of single-step MSC passage 0 (n=6-13), p1 (n=7-12) and p>1 (n=6-12) as well as two-step MSC at p0 (n=6) and p1 (n=8) are shown in Tukey’s Whisker Plots
