# Supplementary material for: GMP-Compliant Isolation and Large-Scale Expansion of Bone Marrow-Derived MSC
Source: PLoS One. 2012 Aug 14;7(8):e43255. doi: 10.1371/journal.pone.0043255 (PMC3419200; doi:10.1371/journal.pone.0043255)
Supplement: Figure S3 — Expression of chondrogenic markers in MSC during differentiation. (DOCX) [file pone.0043255.s003.docx]

**Supplementary Figure S3: Expression of chondrogenic markers in MSC during differentiation.**


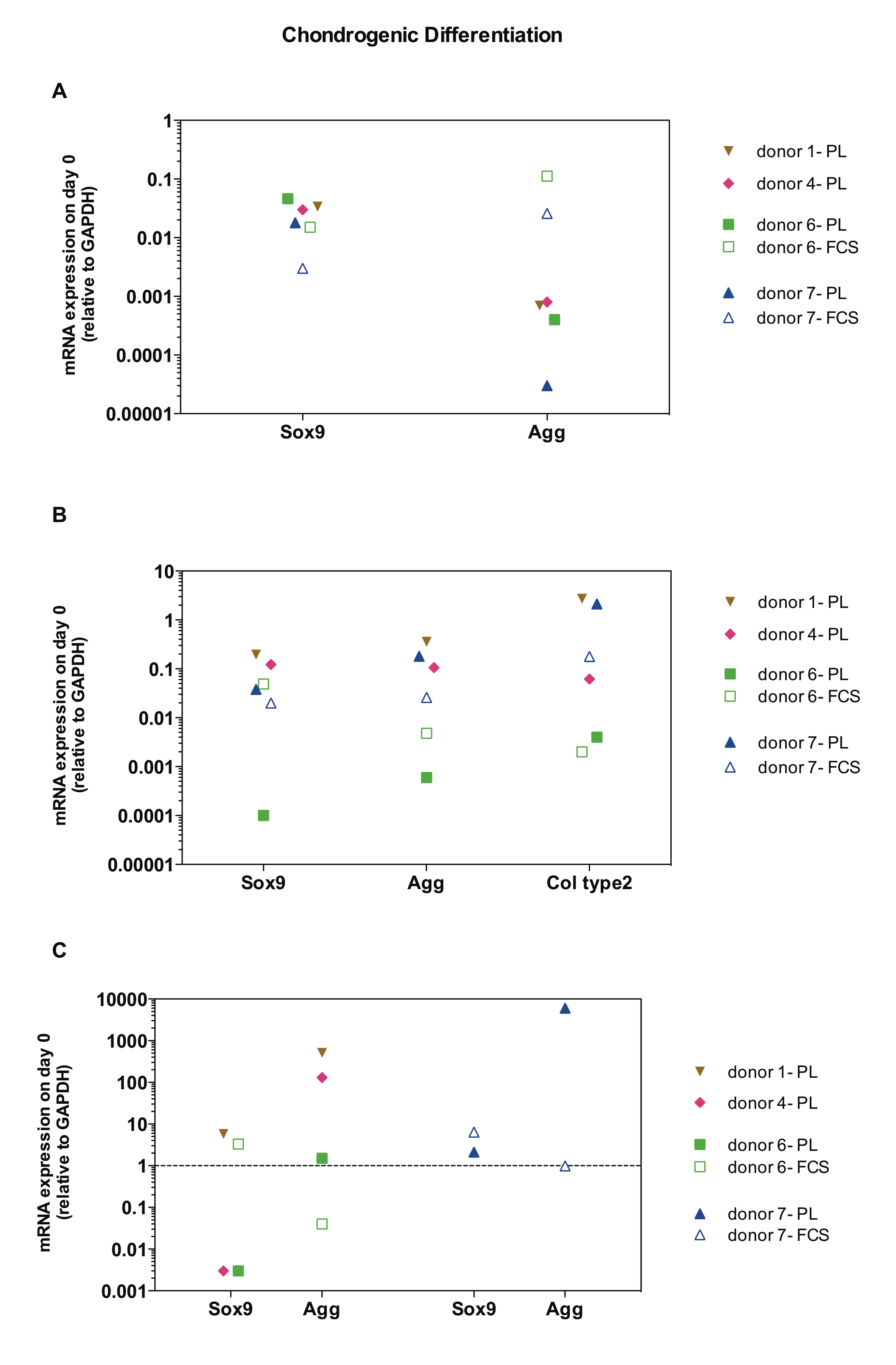


mRNA expression of the transcription factor Sox9 and chondrogenic markers aggrecan (Agg) and collagen type 2 (Col type 2) in pellet culture in chondrogenic conditions was quantitatively measured and normalised to house-keeping gene GAPDH levels. MSC were isolated in complete medium CMSSP supplemented with 10% platelet lysate (PL) or with 10% fetal calf serum (FCS). **A)** relative mRNA expression on day 0. **B)** relative mRNA expression on day 30, **C**) relative mRNA expression on day 30 versus day 0. Dotted line indicates mRNA at day 0.
