# Supplementary material for: GMP-Compliant Isolation and Large-Scale Expansion of Bone Marrow-Derived MSC
Source: PLoS One. 2012 Aug 14;7(8):e43255. doi: 10.1371/journal.pone.0043255 (PMC3419200; doi:10.1371/journal.pone.0043255)
Supplement: Figure S4 — Expression of adipogenic markers in MSC derived from donor 3 during differentiation upon adipogenic conditions. (DOCX) [file pone.0043255.s004.docx]

**Supplementary Figure S4: Expression of adipogenic markers in MSC derived from donor 3 during differentiation upon adipogenic conditions.**


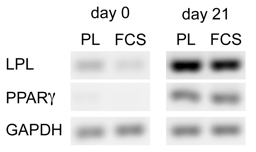


mRNA expression of GAPDH and the differentiation marker genes lipoproteinlipase (LPL) and peroxisome proliferator activated receptor γ (PPARγ) were studied in undifferentiated MSC at day 0 and after differentiation at day 21. MSC were isolated in complete medium CMSSP supplemented with 10% platelet lysate (PL) or with 10% fetal calf serum (FCS).
