# Supplementary material for: GMP-Compliant Isolation and Large-Scale Expansion of Bone Marrow-Derived MSC
Source: PLoS One. 2012 Aug 14;7(8):e43255. doi: 10.1371/journal.pone.0043255 (PMC3419200; doi:10.1371/journal.pone.0043255)
Supplement: Table S1 — Combination of markers used for flow cytometric analysis of MSC cultivated using a GMP-grade single-step or two-step protocol. (DOCX) [file pone.0043255.s005.docx]

**Supplementary Table S1: Combination of markers used for flow cytometric analysis of MSC cultivated using a GMP-grade single-step or two-step protocol.**

**Supplementary Table S1a: Combination of markers for the minimal panel to describe MSC.**

| **No.** | **FITC** | **PE** | **PerCP** |
| --- | --- | --- | --- |
| 1 | IgG | IgG | IgG |
| 2 | CD90 | CD34 | CD45 |
| 3 | CD105 | CD73 | CD3 |
| 4 | HLA- A,B,C | HLA- DR,DQ,DP | - |

**Supplementary Table S1b: Combination of markers for the extended panel.**

| **No.** | **FITC** | **PE** | **PerCP-Cy5.5** | **PE-Cy7** | **APC** |
| --- | --- | --- | --- | --- | --- |
| 1 | IgG | IgG | IgG | IgG | IgG |
| 2 | CD49e | CCR1 | CCR4 | - | CCR3 |
| 3 | - | CCR5 | CCR7 | - | CXCR7 |
| 4 | VEGFR | CD140a | - | CXCR4 | CD140b |
| 5 | CD40 | CCL5 | - | - | CD117 |
| 6 | CD71 | MSCA-1 | - | - | SSEA-4 |
| 7 | CD29 | CD49a | - | - | CD271 |
| 8 | CD200 | CD49c | - | - | CD49d |
| 9 | CD166 | CD146 | CD9 | CD56 | CD31 |
| **No.** | **FITC** | **PE** |  |  | **AlexaFluor 647** |
| 10 | IgG2a (rat) | IgG1 (rat) | - | - | IgM |
| 11 | CD49f | CD51 | - | - | CD349 |
