# Supplementary material for: GMP-Compliant Isolation and Large-Scale Expansion of Bone Marrow-Derived MSC
Source: PLoS One. 2012 Aug 14;7(8):e43255. doi: 10.1371/journal.pone.0043255 (PMC3419200; doi:10.1371/journal.pone.0043255)
Supplement: Table S2 — Characterization of starting material for single-step and two-step cell expansion protocols. (DOCX) [file pone.0043255.s006.docx]

**Supplementary Table S2:** Characterization of starting material for single-step and two-step cell expansion protocols.

|  | **age of donor, years** | **time between collection and culture, [h]** | **cell count, WBC/µL** | **fraction of MNC,%** | **colonies per 10^6^MNC** | **total aspiration volume [mL]** | **total colony content per aspiration** |
| --- | --- | --- | --- | --- | --- | --- | --- |
| **Single-step expansion system - SSP (n = 16)** | | | | | | | |
| **Mean** | 25,5 | 11.8 | 31.2 x 10^3^ | 32.9 | 151 | 31.2 | 40.7 x 10^3^ |
| **S.D.** | 4,4 | 13.8 | 8.89 x 10^3^ | 7.5 | 126 | 13.6 | 36.3 x 10^3^ |
| **Minimum** | 18,0 | 1.0 | 14.7 x 10^3^ | 19.3 | 15 | 10.2 | 1.98 x 10^3^ |
| **Maximum** | 37,0 | 45.0 | 45.7 x 10^3^ | 52.4 | 377 | 54.1 | 139.9 x 10^3^ |
| **Two-step expansion system, option 1 – TSP1 (n = 11)** | | | | | | | |
| **Mean** | 24.5 | 1.7 | 38.1 x 10^3^ | 26.9 | 324 | 25.3 | 84.6 x 10^3^ |
| **S.D.** | 3.1 | 0.6 | 13.9 x 10^3^ | 7.0 | 122 | 5.3 | 51.9 x 10^3^ |
| **Minimum** | 22.0 | 1.0 | 14.0 x 10^3^ | 19.3 | 37 | 17.1 | 6.9 x 10^3^ |
| **Maximum** | 32.0 | 3.0 | 60.9 x 10^3^ | 39.3 | 437 | 36.2 | 196.6 x 10^3^ |
| **Two-step expansion system, option 2 – TSP2 (n = 14)** | | | | | | | |
| **Mean** | 23.8 | 1.8 | 32.7 x 10^3^ | 28.2 | 320 | 27.2 | 80.2 x 10^3^ |
| **S.D.** | 1.8 | 0.6 | 9.7 x 10^3^ | 5.9 | 143 | 8.0 | 48.6 x 10^3^ |
| **Minimum** | 21.0 | 1.0 | 14.0 x 10^3^ | 19.3 | 88 | 17.1 | 16.4 x 10^3^ |
| **Maximum** | 28.0 | 3.0 | 50.2 x 10^3^ | 39.3 | 550 | 42.5 | 160.1 x 10^3^ |
| **Two-step expansion system, option 3 – TSP3 (n = 6)** | | | | | | | |
| **Mean** | 24.5 | 1.7 | 34.3 x 10^3^ | 28.1 | 246 | 23.2 | 53.1 x 10^3^ |
| **S.D.** | 2.2 | 0.8 | 14.0 x 10^3^ | 8.0 | 124 | 4.8 | 37.0 x 10^3^ |
| **Minimum** | 22.0 | 1.0 | 14.0 x 10^3^ | 19.3 | 41 | 17.1 | 7.7 x 10^3^ |
| **Maximum** | 28.0 | 3.0 | 50.2 x 10^3^ | 39.3 | 388 | 30.9 | 109.2 x 10^3^ |
| **Two-step expansion system, option 4 – TSP4 (n = 12)** | | | | | | | |
| **Mean** | 24.2 | 1.6 | 37.0 x 10^3^ | 28.3 | 292 | 25.0 | 80.4 x 10^3^ |
| **S.D.** | 2.1 | 0.7 | 12.8 x 10^3^ | 5.9 | 141 | 7.2 | 59.9 x 10^3^ |
| **Minimum** | 22.0 | 1.0 | 14.0 x 10^3^ | 19.3 | 62 | 17.1 | 11.6 x 10^3^ |
| **Maximum** | 28.0 | 3.0 | 60.9 x 10^3^ | 39.3 | 504 | 42.5 | 189.3 x 10^3^ |

S.D.: standard deviation
