# Supplementary material for: GMP-Compliant Isolation and Large-Scale Expansion of Bone Marrow-Derived MSC
Source: PLoS One. 2012 Aug 14;7(8):e43255. doi: 10.1371/journal.pone.0043255 (PMC3419200; doi:10.1371/journal.pone.0043255)
Supplement: Table S3 — Summary of results for passage 0 for the different expansion protocols. (DOCX) [file pone.0043255.s007.docx]

**Supplementary Table S3:** Summary of results for passage 0 for the different expansion protocols

| **Parameter** | **Overall culture area [cm^2^]** | **Seeding** | | **Days of culture [d]** | **Harvest** | | | |
| --- | --- | --- | --- | --- | --- | --- | --- | --- |
|  |  | **WBC/cm^2^** | **MNC/cm^2^** |  | **MSC/cm^2^** | **Doubling time [h]** | **Population doublings** | **Viability [%]** |
| **Single-step expansion system – SSP (n = 16)** | | | | | | | | |
| **Mean** | 9620 | 38.3 x 10^3^ | 12.0 x 10^3^ | 12.0 | 14.2 x 10^3^ | 22.4 | 12.9 | 93.9 |
| **S.D.** | 7240 | 8.7 x 10^3^ | 0.0 x 10^3^ | 1.5 | 11.6 x 10^3^ | 2.9 | 1.4 | 8.3 |
| **Minimum** | 1272 | 22.9 x 10^3^ | 11.8 x 10^3^ | 9.8 | 0.9 x 10^3^ | 18.8 | 11.1 | 63.9 |
| **Maximum** | 25440 | 62.3 x 10^3^ | 12.2 x 10^3^ | 13.8 | 43.6 x 10^3^ | 29.1 | 15.8 | 100.0 |
| **Two-step expansion system, option 1 – TSP1 (n = 11)** | | | | | | | | |
| **Mean** | 4799 | 50.0 x 10^3^ | 13.4 x 10^3^ | 13.8 | 28.2 x 10^3^ | 26.2 | 12.7 | 96.2 |
| **S.D.** | 3702 | 0.2 x 10^3^ | 3.1 x 10^3^ | 0.1 | 14.3 x 10^3^ | 1.2 | 0.5 | 3.9 |
| **Minimum** | 1272 | 49.3 x 10^3^ | 9.6 x 10^3^ | 13.7 | 6.3 x 10^3^ | 24.8 | 11.7 | 86.2 |
| **Maximum** | 10176 | 50.3 x 10^3^ | 19.4 x 10^3^ | 14.0 | 53.2 x 10^3^ | 28.4 | 13.4 | 100.0 |
| **Two-step expansion system, option 2 – TSP2 (n = 14)** | | | | | | | | |
| **Mean** | 4679 | 50.0 x 10^3^ | 14.1 x 10^3^ | 9.9 | 14.7 x 10^3^ | 20.3 | 11.7 | 95.9 |
| **S.D.** | 2818 | 0.3 x 10^3^ | 2.9 x 10^3^ | 0.1 | 6.9 x 10^3^ | 1.4 | 0.9 | 4.1 |
| **Minimum** | 1272 | 49.3 x 10^3^ | 9.6 x 10^3^ | 9.7 | 3.7 x 10^3^ | 16.9 | 10.5 | 84.1 |
| **Maximum** | 10176 | 50.6 x 10^3^ | 19.4 x 10^3^ | 10.0 | 28.5 x 10^3^ | 22.2 | 14.1 | 100.0 |
| **Two-step expansion system, option 3 – TSP3 (n = 6)** | | | | | | | | |
| **Mean** | 2014 | 50.0 x 10^3^ | 14.0 x 10^3^ | 13.8 | 29.3 x 10^3^ | 25.4 | 13.1 | 92.4 |
| **S.D.** | 845 | 0.3 x 10^3^ | 3.9 x 10^3^ | 0.1 | 21.6 x 10^3^ | 1.5 | 0.8 | 7.4 |
| **Minimum** | 1272 | 49.3 x 10^3^ | 9.6 x 10^3^ | 13.7 | 10.3 x 10^3^ | 23.7 | 12.2 | 78.1 |
| **Maximum** | 3180 | 50.2 x 10^3^ | 19.4 x 10^3^ | 13.9 | 64.9 x 10^3^ | 26.9 | 14.1 | 97.8 |
| **Two-step expansion system, option 4 – TSP4 (n = 12)** | | | | | | | | |
| **Mean** | 2597 | 50.0 x 10^3^ | 14.1 x 10^3^ | 9.9 | 20.8 x 10^3^ | 19.5 | 12.2 | 94.4 |
| **S.D.** | 1637 | 0.3 x 10^3^ | 2.9 x 10^3^ | 0.1 | 14.0 x 10^3^ | 1.7 | 1.1 | 8.5 |
| **Minimum** | 1272 | 49.3 x 10^3^ | 9.6 x 10^3^ | 9.7 | 2.1 x 10^3^ | 16.8 | 10.6 | 79.3 |
| **Maximum** | 5088 | 50.6 x 10^3^ | 19.4 x 10^3^ | 10.0 | 57.0 x 10^3^ | 22.5 | 14.2 | 99.5 |

S.D.: standard deviation
