# Supplementary material for: GMP-Compliant Isolation and Large-Scale Expansion of Bone Marrow-Derived MSC
Source: PLoS One. 2012 Aug 14;7(8):e43255. doi: 10.1371/journal.pone.0043255 (PMC3419200; doi:10.1371/journal.pone.0043255)
Supplement: Table S4 — Summary of results for passage 1 for the different two-step expansion protocol options. (DOCX) [file pone.0043255.s008.docx]

**Supplementary Table S4:** Summary of results for passage 1 for the different two-step expansion protocol options.

| **Parameter** | **Overall culture area [cm^2^]** | **Seeded MSC/cm^2^** | **Days of culture [d]** | **Harvest** | | | |
| --- | --- | --- | --- | --- | --- | --- | --- |
|  |  |  |  | **MSC/cm^2^** | **Doubling time [h]** | **Population doublings** | **Viability [%]** |
| **Two-step expansion system, option 1 – TSP1 (n = 11)** | | | | | | | |
| **Mean** | 6244 | 4.0 x 10^3^ | 6.9 | 41.5 x 10^3^ | 58.5 | 3.2 | 95.8 |
| **S.D.** | 4081 | 0.0 x 10^3^ | 0.3 | 17.3 x 10^3^ | 32.7 | 0.8 | 3.4 |
| **Minimum** | 1272 | 4.0 x 10^3^ | 6.8 | 9.3 x 10^3^ | 39.6 | 1.2 | 86.7 |
| **Maximum** | 10176 | 4.0 x 10^3^ | 7.9 | 72.8 x 10^3^ | 155.4 | 4.2 | 98.6 |
| **Two-step expansion system, option 2 – TSP2 (n = 14)** | | | | | | | |
| **Mean** | 4225 | 4.0 x 10^3^ | 4.8 | 36.9 x 10^3^ | 38.1 | 3.1 | 94.8 |
| **S.D.** | 3343 | 0.0 x 10^3^ | 0.3 | 14.1 x 10^3^ | 7.6 | 0.5 | 5.9 |
| **Minimum** | 1272 | 4.0 x 10^3^ | 3.8 | 18.4 x 10^3^ | 27.6 | 2.2 | 75.0 |
| **Maximum** | 10176 | 4.0 x 10^3^ | 5.0 | 76.9 x 10^3^ | 54.4 | 4.3 | 98.5 |
| **Two-step expansion system, option 3 – TSP3 (n = 6)** | | | | | | | |
| **Mean** | 3392 | 4.0 x 10^3^ | 7.0 | 28.6 x 10^3^ | 67.6 | 2.7 | 95.0 |
| **S.D.** | 2077 | 0.0 x 10^3^ | 0.4 | 18.0 x 10^3^ | 20.4 | 0.7 | 3.8 |
| **Minimum** | 1272 | 4.0 x 10^3^ | 6.8 | 14.3 x 10^3^ | 40.9 | 1.8 | 87.6 |
| **Maximum** | 6360 | 4.0 x 10^3^ | 7.9 | 64.2 x 10^3^ | 102.9 | 4.0 | 97.4 |
| **Two-step expansion system, option 4 – TSP4 (n = 12)** | | | | | | | |
| **Mean** | 2809 | 4.0 x 10^3^ | 4.9 | 44.4 x 10^3^ | 36.9 | 3.3 | 96.0 |
| **S.D.** | 1788 | 0.0 x 10^3^ | 0.5 | 17.9 x 10^3^ | 8.8 | 0.7 | 2.1 |
| **Minimum** | 1272 | 4.0 x 10^3^ | 3.8 | 12.1 x 10^3^ | 28.5 | 1.6 | 92.5 |
| **Maximum** | 6360 | 4.0 x 10^3^ | 5.9 | 73.8 x 10^3^ | 56.4 | 4.2 | 99.3 |

S.D.: standard deviation
