# Supplementary material for: GMP-Compliant Isolation and Large-Scale Expansion of Bone Marrow-Derived MSC
Source: PLoS One. 2012 Aug 14;7(8):e43255. doi: 10.1371/journal.pone.0043255 (PMC3419200; doi:10.1371/journal.pone.0043255)
Supplement: Table S5 — Calculations on the potential MSC-yield for the different expansion systems. (DOCX) [file pone.0043255.s009.docx]

**Supplementary Table S5:** Calculations on the potential MSC-yield for the different expansion systems.

|  | **Passage 0** | | **Passage 1** | |
| --- | --- | --- | --- | --- |
| **Parameter** | **MSC harvested/µL BM seeded** | **MSC harvested/BM-aspiration** | **MSC harvested/µL BM seeded** | **MSC harvested/BM-aspiration** |
| **Single-step expansion system (n = 16) – SSP** | | | | |
| **Mean** | 11.9 x 10^3^ | 356.1 x 10^6^ | n/a | n/a |
| **S.D.** | 9.1 x 10^3^ | 426.7 x 10^6^ | n/a | n/a |
| **Minimum** | 0.3 x 10^3^ | 9.4 x 10^6^ | n/a | n/a |
| **Maximum** | 33.8 x 10^3^ | 1828.8 x 10^6^ | n/a | n/a |
| **Two-step expansion system, option 1 (n = 11) – TSP1** | | | | |
| **Mean** | 21.2 x 10^3^ | 554.1 x 10^6^ | 235.3 x 10^3^ | 6279.9 x 10^6^ |
| **S.D.** | 12.9 x 10^3^ | 368.6 x 10^6^ | 175.4 x 10^3^ | 5100.1 x 10^6^ |
| **Minimum** | 2.9 x 10^3^ | 65.6 x 10^6^ | 6.6 x 10^3^ | 152.9 x 10^6^ |
| **Maximum** | 44.7 x 10^3^ | 1245.1 x 10^6^ | 591.3 x 10^3^ | 15520.6 x 10^6^ |
| **Two-step expansion system, option 2 (n = 14) – TSP2** | | | | |
| **Mean** | 9.6 x 10^3^ | 261.1 x 10^6^ | 91.5 x 10^3^ | 2508.8 x 10^6^ |
| **S.D.** | 4.7 x 10^3^ | 138.1 x 10^6^ | 76.1 x 10^3^ | 2321.9 x 10^6^ |
| **Minimum** | 1.7 x 10^3^ | 38.4 x 10^6^ | 16.4 x 10^3^ | 377.8 x 10^6^ |
| **Maximum** | 16.4 x 10^3^ | 507.7 x 10^6^ | 315.7 x 10^3^ | 9753.7 x 10^6^ |
| **Two-step expansion system, option 3 (n = 6) – TSP3** | | | | |
| **Mean** | 19.8 x 10^3^ | 482.8 x 10^6^ | 214.3 x 10^3^ | 5424.2 x 10^6^ |
| **S.D.** | 19.6 x 10^3^ | 532.5 x 10^6^ | 360.6 x 10^3^ | 9565.8 x 10^6^ |
| **Minimum** | 5.8 x 10^3^ | 132.8 x 10^6^ | 20.7 x 10^3^ | 476.3 x 10^6^ |
| **Maximum** | 59.0 x 10^3^ | 1549.0 x 10^6^ | 947.8 x 10^3^ | 24879.3 x 10^6^ |
| **Two-step expansion system, option 4 (n = 12) – TSP4** | | | | |
| **Mean** | 15.2 x 10^3^ | 394.4.3 x 10^6^ | 173.8 x 10^3^ | 4487.3 x 10^6^ |
| **S.D.** | 9.1 x 10^3^ | 272.3 x 10^6^ | 151.9 x 10^3^ | 4479.1 x 10^6^ |
| **Minimum** | 1.0 x 10^3^ | 22.2 x 10^6^ | 11.6 x 10^3^ | 267.8 x 10^6^ |
| **Maximum** | 30.3 x 10^3^ | 834.9 x 10^6^ | 550.2 x 10^3^ | 15406.8 x 10^6^ |

S.D.: standard deviation. n/a: not applicable
